# 

**Published:** 1857-04

**Authors:** 


### CONTENTS.

### ORIGINAL COMMUNICATIONS.

PAGE

ARTICLE I.—Foreign Correspondence. By W. F. WESTMORELAND, M. D., Professor of the Principles and Practice of Surgery in the Atlanta Med. College,........ 449

ARTICLE II.—Scarlet Fever. By REUBEN SEARCY, M. D., Tuscaloosa, Ala., 259

ARTICLE III.—Essay. By THOMAS S. DENNY, M. D., read before the Atlanta Medical Society, and published by that body,........ 463

ARTICLE IV.—Report of a case of Hemorrhage from the Lungs in a child 3 years of age. By HAYDEN COE, M. D., Atlanta, Ga.,........ 467

### SELECTIONS.

"Moral Insanity,"........ 470

Letter from Boston,........ 472

Sketch of the Geology of Tennessee,........ 478

A Monagraph on Ovarian Tumors; with an extended view of Ovariotomy as a means of cure,........ 481

The Presidency, and place of Meeting of the American Medical Association, 489

The Medical Testimony of Drs. Willard Parker and Gilman in the Trial of Huntington,........ 491

On the Value of Quinine as a Therapeutic Agent,........ 494

Intemperance in Europe and the East,........ 497

### EDITORIAL AND MISCELLANEOUS.

The Next Session,........ 502

American Medical Association,........ 502

Medical Society of Georgia,........ 503

"The Southern Journal of the Medical and Physical Sciences,"........ 503

Medical College of the State of South Carolina,........ 504

American Medical Association,........ 504

A Convention of Medical Editors,........ 506

Effects of Chloroform on Uterine Contractions,........ 507

Dr. Livingston,........ 508

Contest between Mr. Fergusson and Mr. Syme,........ 509

When is a Medical Teacher or Practitioner Superannuated?........ 511

Leeches,........ 511

Researches on the Cause of the Fluidity of the Blood,........ 511

Subscriptions Received,........ 512

Meteorological Observations for March, 1857, Atlanta, Ga.,........ 512

